# Presence of Sugar in the Liver

**Published:** 1849-03

**Authors:** 


*Presence of Sugar in the Liver.*—MM. Bernard and Barreswill have demonstrated the presence of a notable quantity of sugar in the liver both of man and animals. By fermentation they obtained alcohol from this sugar, a sample of which M. Pelouze exhibited to the Academy. Hitherto no means had been ascertained of obtaining from the liver other than a kind of molasses charged with salts, the sugar of which was uncrystallized. Repeated experiments have enabled MM. Bernard and Barreswill to establish the fact that the sugar, which exists in considerable proportion in the tissue of the liver, is not found in a normal state in any other organ, and that conseqently the liver is, on this account, chemically distinguishable from all the other organs of the animal economy. They have satisfied themselves that the liver always contains the same large proportion of sugar, even in animals completely deprived of food containing either sugar or starch, and kept for a long time exclusively on animal diet. They conclude that the existence of sugar in the liver is a physiological fact completely independent of the kind of food taken.—*London Medical Gazette.—Ibid.*

